# Exercise or not? An empirical illustration of the role of behavioral alternatives in exercise motivation and resulting theoretical considerations

**DOI:** 10.3389/fpsyg.2023.1049356

**Published:** 2023-02-01

**Authors:** Sinika Timme, Ralf Brand, Michaela Raboldt

**Affiliations:** Sport and Exercise Psychology, University of Potsdam, Potsdam, Germany

**Keywords:** eye-tracking, dual-process models, situated processes, motivation, physical activity

## Abstract

**Objective:**

Individuals’ decisions to engage in exercise are often the result of in-the-moment choices between exercise and a competing behavioral alternative. The purpose of this study was to investigate processes that occur in-the-moment (i.e., situated processes) when individuals are faced with the choice between exercise and a behavioral alternative during a computerized task. These were analyzed against the background of interindividual differences in individuals’ automatic valuation and controlled evaluation of exercise.

**Method:**

In a behavioral alternatives task 101 participants were asked whether they would rather choose an exercise option or a behavioral alternative in 25 trials. Participants’ gaze behavior (first gaze and fixations) was recorded using eye-tracking. An exercise-specific affect misattribution procedure (AMP) was used to assess participants’ automatic valuation of exercise before the task. After the task, self-reported feelings towards exercise (controlled evaluation) and usual weekly exercise volume were assessed. Mixed effects models with random effects for subjects and trials were used for data analysis.

**Results:**

Choosing exercise was positively correlated with individuals’ automatic valuation (*r* = 0.20, *p* = 0.05), controlled evaluation (*r* = 0.58, *p* < 0.001), and their weekly exercise volume (*r* = 0.43, *p* < 0.001). Participants showed no bias in their initial gaze or number of fixations towards the exercise or the non-exercise alternative. However, participants were 1.30 times more likely to fixate on the chosen alternative first and more frequently, but this gaze behavior was not related to individuals’ automatic valuation, controlled evaluation, or weekly exercise volume.

**Conclusion:**

The results suggest that situated processes arising from defined behavioral alternatives may be independent of individuals’ general preferences. Despite one’s best general intention to exercise more, the choice of a non-exercise alternative behavior may seem more appealing in-the-moment and eventually be chosen. New psychological theories of health behavior change should therefore better consider the role of potentially conflicting alternatives when it comes to initiating physical activity or exercise.

## 1. Introduction

Promoting exercise is one of the most critical public health priorities, considering being insufficiently active increases the risk of death by 20–30% compared to being sufficiently active (World Health Organization, 2020). Understanding the psychological processes that guide the choice to be physically active is key to more effectively promoting regular exercise behavior. In the past 20 years, exercise psychology has been largely dominated by a focus on social-cognitive and humanistic/organismic frameworks that conceptualize behavior change as a mostly unidirectional process, such that a behavior is done based on mentally imagined goals (e.g., the idea of going for a run, which may have positive consequences or fit particularly well with our subjective values; Rhodes et al., 2019; Ekkekakis and Brand, 2021). This framework is based on the assumption that individuals form expectations (e.g., that exercise is important and doable) from which the intention to exercise culminates (Rhodes et al., 2019). Intention as a primary antecedent of behavior is one of the cornerstones of the social-cognitive framework, yet empirical evidence reveals a consistent intention-behavior gap (Rhodes and de Bruijn, 2013). Possible reasons for this gap are negative exercise-related automatic tendencies that are contrary to the intention (Brand and Ekkekakis, 2021), such as negative automatic associations (Schinkoeth and Antoniewicz, 2017), affective valuations (Schinkoeth and Brand, 2020), habit or identity (Rhodes, 2017, 2021).

Only recently, dual-process models that emphasize the role of automatic processes in addition to controlled cognitive processes (e.g., forming an intention from expectations about the future), have been applied to exercise psychology. According to a recent review, dual-process models are ‘the most recent and understudied framework for understanding physical activity’ (Rhodes et al., 2019, p. 100). Moreover, there is at least one other characteristic of dual-process models that needs to be emphasized. The dual-process framework implies that automatically activated momentary processes are essentially predetermined by the situation and therefore also referred to as *situated* processes (Brand and Ekkekakis, 2018). They may conflict with behavioral plans and must be analyzed in terms of their importance for behavioral regulation.

Examples of dual-process theories that address the role of *situated processes* within exercise and physical activity behavior include the Affective-Reflective Theory of Physical Inactivity and Exercise (ART; Brand and Ekkekakis, 2018) and the Theory of Effort Minimization in Physical Activity (TEMPA; Cheval and Boisgontier, 2021). The two have been recently contrasted in a theoretical article with an argument that provides the foundation for the current study (Brand and Cheval, 2019). Both theories are grounded in the idea that in-the-moment when individuals have to make a choice between one behavior (e.g., do exercise) or a competing behavioral alternative (e.g., remain physically inactive), a *momentary conflict* may arise before a choice is made. According to the ART, there are situated automatic affective processes that have been learned through previous experiences with exercise that can prevent individuals from rationally considering becoming physically active (a negative affective valuation of exercise) or steer us toward it (a positive affective valuation of the behavior). The TEMPA assumes that a hard-wired evolutionary process is default, which accounts for an ever-present behavioral tendency to avoid and economize physical activity and may conflict with more rational considerations.

Multiple experimental studies support the perspective of dual-process theories that when individuals are confronted with an exercise-related stimulus an immediate psychological response (e.g., affective reaction or approach/avoidance tendency) is triggered (Rebar et al., 2016; Schinkoeth and Antoniewicz, 2017). Previous studies have typically measured automatic (e.g., Chevance et al., 2017) and controlled processes first (e.g., Kiviniemi et al., 2007), which were then either correlated with remembered usual exercise behavior (e.g., Bluemke et al., 2010) or used to predict exercise behavior in subsequent weeks (e.g., Antoniewicz and Brand, 2016). Findings from these studies suggest that those who are more active tend to focus more on exercise stimuli. Despite previous literature on interindividual differences (e.g., automatic processes) and distal behavior outcomes (e.g., usual exercise volume), less is known about potentially conflicting situated processes that occur in-the-moment an individual is asked to choose a behavior. For example, some may have a strong automatic preference for exercise, but when confronted with a competing non-exercise behavioral option, the behavioral alternative may seem even more attractive in that particular moment and eventually be chosen.

Harris and Bray (2019, 2021) examined single situated exercise decisions. Participants had to choose between an exercise vs. a non-exercise task (e.g., seated “free time” with smartphone) after completing either a high-or low-cognitive demand task. The high cognitive demand task resulted in increased mental fatigue, which in turn decreased likelihood of choosing to exercise. These findings emphasize the importance of situated factors (e.g., mental fatigue) in an individual’s in-the-moment choice whether or not to exercise.

In a recent study, Cheval et al. (2020) took situated processes into account by employing a paradigm in which eye-tracking was used to examine participants’ gaze behavior while they viewed mutually exclusive behaviors. The authors found that physically active individuals were generally more likely to focus their attention on physical activity stimuli than on stimuli representing a sedentary alternative.

The study presented here builds on these findings, but examines situated gaze in a more complete behavioral situation: We monitored participants’ gaze behavior when they have to *choose* between an exercise-related stimulus and a stimulus displaying a non-exercise alternative, and analyze their choices on the background of previously measured interindividual differences in self-reported exercise behavior, automatic valuation of exercise and self-reported feelings towards exercise.

In other fields, such as consumer psychology, process tracing methods are frequently used to capture situated processes in order to assess which factors play a role during behavioral decision-making (e.g., information search strategy). For example, eye-tracking has often been used to assess attentional processes during behavioral or consumer choices. Commonly used measures are *first gaze* (i.e., first fixated location) and number of *fixations* (i.e., temporally closely spaced fixated locations for a period of time). First gaze has shown a weak and inconsistent association with choice behavior. Schotter et al. (2010) demonstrated that participants were slightly more likely to choose the item they fixated on first. In contrast, Krajbich et al. (2010) found that the probability of fixating an item first was unaffected by their initially preferred ratings. A more homogenous pattern of results emerges for number of fixations. Previous research supports the idea that the more time we spend on an item, the more likely we are to choose it (Krajbich et al., 2010; Cavanagh et al., 2014). However, researchers disagree on whether this relation is causal, leaving open the question of whether we direct our attention on what we like or we will like what we focus our attention on (Orquin and Mueller Loose, 2013).

The present study aimed to extend insights on the processes occurring when individuals are confronted with competing behavioral alternatives. We administered eye-tracking in a computerized task where participants were asked to choose between an exercise and a non-exercise alternative in a series of hypothetical situations. Gaze behavior was tracked to examine how much attention was paid to each behavioral alternative in each situation of choice. This allowed us to measure both interindividual (e.g., who is generally more likely to look at exercise) and intraindividual processes (e.g., which of the behavioral alternatives is more likely to be fixated) and use them as proxies for situated processes that would likely occur in real life situations.

According to the TEMPA, one could assume an initial bias towards the non-exercise alternative (Cheval and Boisgontier, 2021). With the ART conceptualizing the automatic response as a learned process (Brand and Ekkekakis, 2018) one would assume that individuals who (have learned to like and do) exercise more regularly will have an initial bias towards the exercise alternative. Based on findings from consumer psychology, we expected that individuals would be more likely to initially direct their gaze toward the chosen alternative and fixate this alternative more often. Whilst the current study emphasized the examination of gaze behavior as situated processes within individuals, we recognize that interindividual differences in automatic and controlled processes are also relevant to exercise behavior (e.g., Schinkoeth and Antoniewicz, 2017; Rhodes et al., 2019). In line with the constructs of the ART (Brand and Ekkekakis, 2018), we included analyses of the association of automatic valuation of exercise, self-reported feelings towards exercise (controlled evaluation) and exercise behavior with gaze behavior on a subject-level as well. Based on previous findings (Cheval et al., 2020) we expect individuals with higher levels of self-reported exercise behavior (and more positive automatic and controlled (e)valuations of exercise) to display higher attentional focus (first gaze and fixations) on exercise-related stimuli. By simultaneously considering inter-and intraindividual varying processes when individuals are confronted with exercise-related choices, this study introduces a new approach to investigate situated processes in exercise psychology.

## 2. Materials and methods

### 2.1. Participants

106 students from the University of Potsdam took part in this study. Participants were recruited through the university’s participant pool. Five participants were removed from the analysis due to technical problems during data collection, resulting in a total sample of *N* = 101 participants (*M*_age_ = 23.6, *SD*_age_ = 3.6, 48.5% females). Most of the participants were enrolled in a sports science (*n* = 80) or psychology (*n* = 21) program. All participants provided written consent before the experiment, fulfilled the screening criteria (i.e., no confounding activities such as intensive exercise or alcoholic beverages beforehand), and reported having a normal or corrected-to-normal vision without color blindness. Participants were compensated for their participation with additional (non-obligatory) course credit. The study was conducted following the ethical standards laid out in the Declaration of Helsinki and the local institution’s ethical guidelines. Data, analysis code, and stimulus material are available.^1^

### 2.2. Measures

#### 2.2.1. Behavioral alternatives task

**Figure 1:**
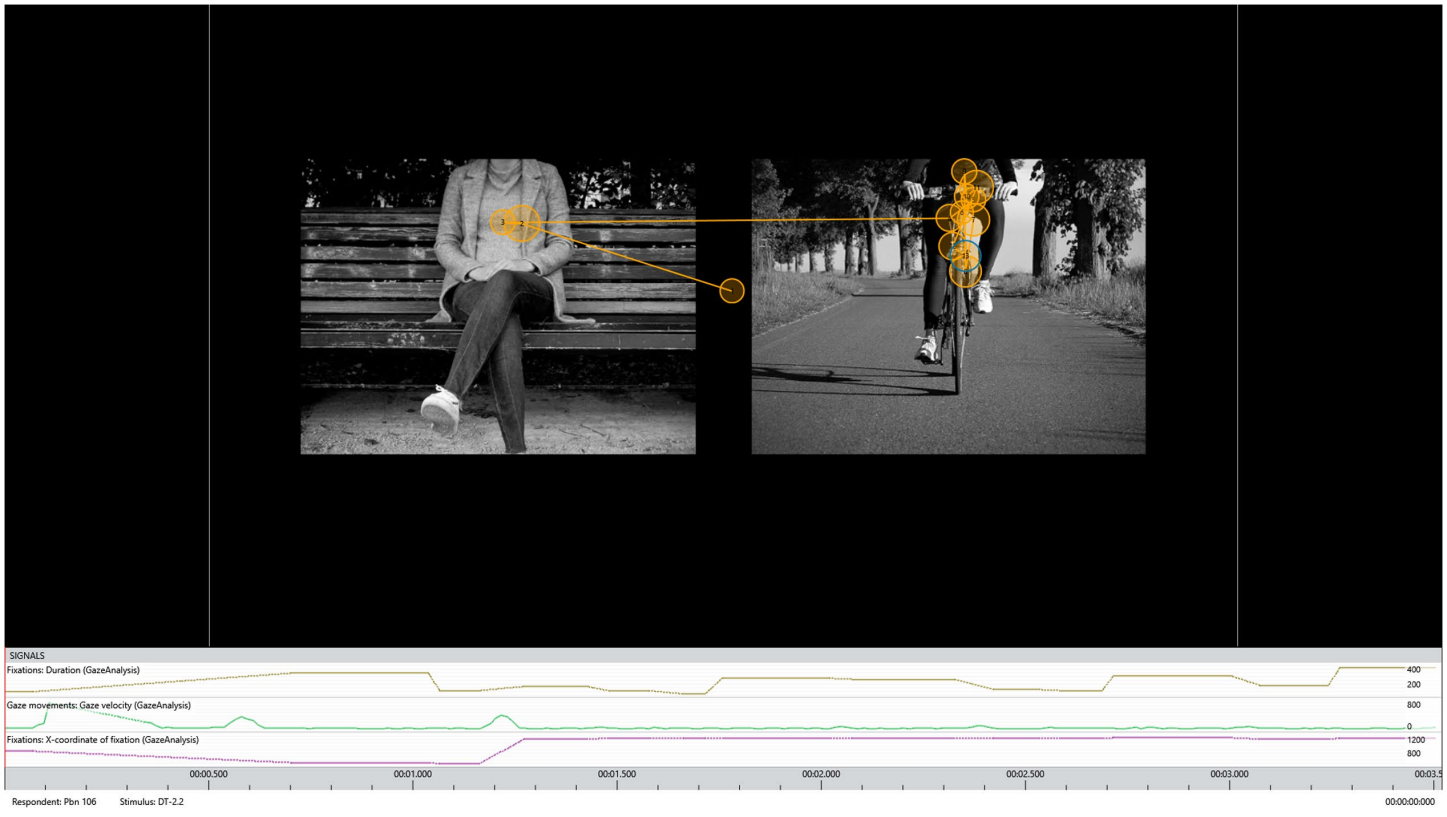
Representative gaze replay of a single participant with circles representing fixations and lines representing saccades. Fixation duration is indicated by circle size. Screenshot reproduced with permission from iMotions (8.0).

For the behavioral alternatives task, we adapted the idea of the Situated Decisions to Exercise Questionnaire (SDEQ) by Brand and Schweizer (2015) in a computerized task presented with iMotions^™^ software (version 8.0). After reading a prototypical everyday situation (vignette; e.g., a friend has asked you if you would either like to work out with him tonight or have a lazy evening), five randomized pairs of pictures representing conflicting behavioral alternatives (exercise vs. non-exercise) were presented. In each of the trials, participants were forced to choose one of the presented behavioral alternatives they would engage in (see Figure 1). Two vignettes each described situations where the activities would be done alone (vignettes 1 and 5) or together with others (vignettes 2 and 3), respectively. One vignette described an ambivalent situation where the individual could choose to do the behavior alone or in a group (vignette 4). Thus, participants completed 5 vignettes with 5 randomized pairs of pictures resulting in a total of 25 trials. The pictures were presented side-by-side on the left and right sides of the computer screen. The side of the screen was randomized for the exercise and non-exercise alternative. Choices had to be made within 10 s by clicking on either the ‘E’ (left behavioral alternative) or ‘I’ (right behavioral alternative) button on a keyboard.

A 10-s time constraint with manual advance was set. To prevent participants from engaging excessively in deliberate thinking, they were asked to choose based on their initial thought as fast as possible. Between the trials, participants had to focus on a fixation cross for 5 s.

In total, 50 different pictures were used in the task: 25 representing exercise and 25 representing non-exercise. The exercise activities were selected according to the results of a representative survey on common sports and exercise activities among the Berlin population (Dierker et al., 2018). The results of that survey indicated biking, running, fitness, swimming, and hiking as the five most frequent activities. Since primarily moderate-or vigorous-intensity activities should be displayed in the current study, hiking was not considered; however, additional fitness activities were included based on exercise trends (e.g., CrossFit, rollerblading). For the non-exercise alternative, a broad range of alternatives were selected such as reading, listening to music, and lying in the park. Images were mainly provided by a license-free image database,^2^ and four images were self-taken by the authors. All images were presented in grayscale (16 bit) with a minimum resolution of 1,024 × 768 pixels and processed so that brightness distribution and contrasts were matched. The exercise and non-exercise images had to fulfill the following requirements: a similar perspective, the same number of individuals on the images with no visible facial expressions, no sexual stimuli, and no labels.

Intraindividual differences in gaze behavior and choice behavior for the behavioral alternatives were repeatedly measured and analyzed for each choice trial during the task. Since these measures can differ from situation to situation within individuals, they were used as a proxy for situated processes.

##### 2.2.1.1. Gaze behavior

Gaze behavior (*first gaze* and *fixations*) was measured with the Gazepoint GP3 eye-tracker at a sampling frequency of 60 Hz. For each trial, a *first gaze* toward the exercising picture was coded as 1, whereas a first gaze toward the non-exercise picture was coded as 0. *Fixations* are a period during which the eyes are locked on a specific location in the visual field, measured by the eye tracker as a series of very close gaze points in time and range. The I-VT algorithm was used to classify eye movements above the velocity threshold of 30°/s as a fixation (Olsen, 2012). Number of fixations was separately computed for the exercising and the non-exercise alternative.

##### 2.2.1.2. Choice

For each trial, choosing the exercise alternative (*choice*) was coded as 1, whereas choosing the non-exercise alternative was coded as 0.

#### 2.2.2. Interindividual differences

Interindividual differences in participants’ automatic valuation of exercise was assessed before the task, whereas self-reported feelings towards exercise and exercise behavior were assessed after completing the task.

##### 2.2.2.1. Automatic valuation of exercise

The affective misattribution procedure (AMP; Payne et al., 2005) was used as a proxy for an automatic-affective valuation of exercise. The AMP uses supraliminal presentations of primes (of the affective target stimuli, e.g., exercise) followed by a neutral Chinese ideograph. It is assumed that participants misattribute their spontaneous affective response to the primes for evaluation of the Chinese ideographs (Payne and Lundberg, 2014). In this study, an adapted version of the standard AMP (Payne et al., 2005) was presented with Inquisit 5.0 software. The same exercise and non-exercise pictures from the behavioral alternatives task were used as target primes, and grey squares were used as neutral primes. Primes were presented for 75 ms followed by a 125 ms black screen and by the presentation of the Chinese ideograph for 200 ms. Then, a grey mask picture was shown until participants evaluated the ideograph as “pleasant” or “unpleasant” by pressing the “E” or “I” key, respectively, on a standard QWERTZ keyboard. Participants were instructed to ignore the prime stimulus (Payne and Lundberg, 2014) and completed 100 randomly presented trials, lasting approximately five minutes total. The AMP score was calculated as the difference between the proportions of ideographs evaluated as pleasant after the exercise primes vs. the non-exercise primes divided by 100, resulting in a score between -1 and 1 (Payne et al., 2005). Positives scores indicated more ideographs following an exercise prime were evaluated as pleasant, whereas negatives scores indicated more ideograph following a non-exercise prime were evaluated as pleasant. The AMP score was z-transformed before further analyses. The internal consistency of the AMP in this sample (split-half; *p* = 0.81) is similar to that found in previous studies (>0.80; e.g., Zenko and Ekkekakis, 2019). We chose the AMP score as an implicit measure of automatic-affective valuation of exercise due to its inherent core affective and valuative properties. The AMP is based on the theoretical idea to elicit a spontaneous, automatic, affective judgement. This is conceptually close to the construct of automatic-affective valuation of exercise according to the ART (Brand and Ekkekakis, 2018; in contrast, for example, implicit association tests are based much more on the assumption of mental representations). Many studies from different research areas have already used the AMP to draw conclusions about automatic affective reactions to a wide range of behaviors, including drinking decisions (Payne et al., 2008), moral decisions (Hofmann and Baumert, 2010) and eating behavior (e.g., Hofmann et al., 2010). According to a meta-analysis (Cameron et al., 2012), the AMP can be used to predict behavior with an average effect size of *r* = 0.35. Few original studies in exercise psychology have used the AMP, but had comparable results (Karpen et al., 2012; Antoniewicz and Brand, 2014).

##### 2.2.2.2. Self-reported feelings towards exercise

Self-reported feelings associated with exercise was used as a proxy for controlled evaluation of exercise. Participants indicated how they felt about exercising on a continuous 7-point scale (“absolutely negative” to “absolutely positive”). Scores for self-reported positive feelings were z-standardized. Research has shown that single-item measures to capture exercise-related feelings are highly correlated with multi-item measured of the same construct (*r* = 0.56 to 0.70; Brito et al., 2022).

##### 2.2.2.3. Self-reported exercise volume

Self-reported exercise volume was measured through questions from the International Physical Activity Questionnaire (short form; Craig et al., 2003) as a proxy for a behavioral component. Participants were asked about their usual exercise behavior in their free time. Exercising was defined as activities that are deliberately pursued in a way that makes one breathe faster and break a sweat (e.g., swimming, jogging, going to the gym, tennis, soccer). Participants indicated their weekly frequency and duration of exercise sessions according to this definition. Average weekly exercise volume (sessions per week × duration per session) was calculated. One participant who reported an average duration of 360 minutes per session was excluded from the analyses involving self-reported exercise volume but retained for all other analyses.

### 2.3. Procedure

Participants were tested in single-person lab sessions lasting for approximately 45 min. The laboratory was dimmed with artificial lightning (i.e., no sunlight). Participants were seated 60 cm in front of a Benq Senseq FP222WA, 22″ monitor. The monitor was connected to the investigator’s laptop. The investigator could thereby monitor the experiment, but was out of the participant’s sight.

First, participants completed the AMP and then manually advanced to the behavioral alternatives task. Before initiating behavioral alternatives task, calibration of the screen-based Gazepoint eye-tracker was done by the iMotions^™^ software. Participants were instructed to minimize head movements during eye-tracking recording. After successful calibration, participants completed the behavioral alternatives task. After the task, participants answered a follow-up questionnaire to control for possible confounders (e.g., excessive exercise before the experiment, demographics) and to assess the exercise-related controlled and behavioral component. Finally, participants were thanked and debriefed.

### 2.4. Data analysis

The data were analyzed using generalized mixed models with the lme4-package (Bates et al., 2015) in R-software (R Core Team, 2021). Logistic mixed-effects models were used to predict the odds of first gaze (exercise vs. non-exercise) and linear mixed-effects models to predict the number of fixations on the behavioral alternatives. Participants and trials were included as crossed random effects to account for the crossed data structure and the non-independence of observations. Assuming a medium sized effect (based on a meta-analysis on the effect of visual attention on choice; Bhatnagar and Orquin, 2022), simulation studies revealed that in a fully crossed design with 25 trials 90 participants or more would result into 80% power (Westfall et al., 2014). To account for study attrition and data loss we aimed for a sample of at least 100 participants.

First, unconditional means models with the respective dependent variable (first gaze, exercise fixations, non-exercise fixation) were computed. Second, choice (0 = non-exercise, 1 = exercise) was added to model to test the relationship between gaze and choice behavior. Third, interindividual variables (i.e., automatic valuation of exercise, self-reported feelings towards exercise, and self-reported exercise volume) were separately introduced into the models to examine interindividual differences in gaze behavior.

## 3. Results

### 3.1. Choices in the behavioral alternatives task

In the behavioral alternatives task, choosing the exercise alternative was more likely than choosing the non-exercise alternative (*OR* = 1.85, 95% CI [1.39; 2.47], *p* < 0.001). In other words, there was a 65% chance of choosing exercise across all trials and participants. Choosing the exercise alternative in the behavioral alternatives task correlated with self-reported exercise volume (*r* = 0.43, 95% CI [0.20, 0.53]*, p* < 0.001), with self-reported positive feelings towards exercise (*r* = 0.58, 95% CI [0.43, 0.70]*, p* < 0.001) and with the automatic valuation of exercise as measured with the AMP (*r* = 0.20, 95% CI [0.00, 0.38], *p* = 0.05). Correlations and descriptive statistics of all main variables are presented in Table 1.

**Table 1:** Means, standard deviations, and correlations of the main variables.

Variable	*M*	*SD*	1	2	3	4	5	6
(1) Exercise volume	358.36	283.39						
(2) Controlled evaluation	6.33	0.86	0.43**					
			[0.26, 0.58]					
(3) Automatic-affective valuation	0.02	0.17	0.15	0.17				
			[−0.04, 0.34]	[−0.03, 0.35]				
(4) First gaze (exercise)	13.68	2.06	0.14	0.01	−0.06			
			[−0.05, 0.33]	[−0.19, 0.21]	[−0.25, 0.14]			
(5) Exercise fixations	4.00	2.67	−0.02	−0.07	−0.10	0.16		
			[−0.22, 0.18]	[−0.26, 0.13]	[−0.29, 0.09]	[−0.03, 0.35]		
(6) Nonexercise fixations	3.92	2.73	−0.04	−0.23*	−0.13	0.04	0.82**	
			[−0.23, 0.15]	[−0.41, −0.04]	[−0.32, 0.07]	[−0.16, 0.23]	[0.74, 0.88]	
(7) General decision tendency	0.62	0.20	0.43**	0.58**	0.20*	0.09	−0.13*	−0.34**
			[0.25, 0.58]	[0.43, 0.70]	[0.00, 0.38]	[−0.11, 0.28]	[−0.31, −0.07]	[−0.50, −15]

M and SD are used to represent mean and standard deviation, respectively. Values in square brackets indicate the 95% confidence interval for each correlation. * indicates *p* < 0.05. ** indicates *p* < 0.01.

### 3.2. Gaze behavior

#### 3.2.1. First gaze

There was no significant difference in whether participants fixated the exercise or the non-exercise alternative first (*OR* = 1.29, 95% CI [0.89, 1.88], *p* = 0.18), suggesting there was no initial bias towards the non-exercise alternative. However, the initial gaze fixation was more likely on the alternative that was then chosen by the participant (*OR* = 1.30, 95% CI [1.04, 1.62], *p* = 0.02). Self-reported exercise volume (*OR* = 1.00, 95% CI [1.00, 1.00], *p* = 0.39), self-reported positive feelings towards exercise (*OR* = 0.99, 95% CI [0.90, 1.08], *p* = 0.77), and automatic valuation of exercise (*OR* = 0.98, 95% CI [0.89, 1.07], *p* = 0.66) did not contribute significantly to explaining variance in first gaze.

#### 3.2.2. Fixations

**Figure 2:**
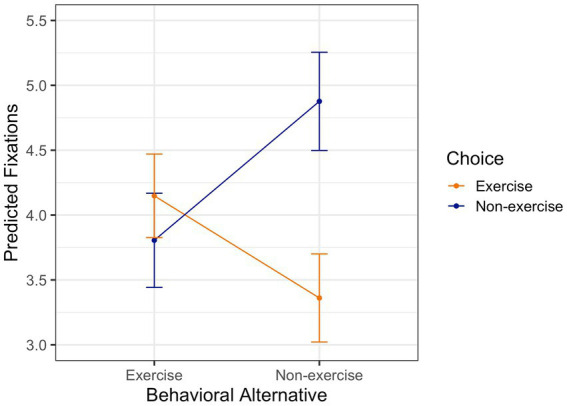
Predicting fixations on the exercise and the non-exercise behavioral alternative with choice behavior. The orange line shows the model-predicted fixations when exercise was chosen. The blue line shows the model predicted fixations when non-exercise was chosen.

Analyses revealed that individuals had more fixations on the chosen alternative compared to the non-chosen alternative (*b*_non-ex_ = 1.07, 95% CI [0.78, 1.36], *p* < 0.001, *b*_ex_ = −0.79, 95% CI [−1.05, −0.53], *p* < 0.001). Figure 2 illustrates this effect, showing participants had more fixations on non-exercise (compared to exercise) when choosing non-exercise (orange line) and more fixations on exercise (compared to non-exercise) when choosing exercise (blue line). In each trial, exercise was fixated on average 3.99 times (95% CI [3.66, 4.31]) and non-exercise 3.90 times (95% CI [3.56, 4.24]) before one of the two alternatives were selected. There was no significant difference in the number of fixations on the exercise vs. the non-exercise alternative accordingly (*b* = −0.09, 95% CI [−0.34, 0.17], *p* = 0.51).

**Figure 3:**
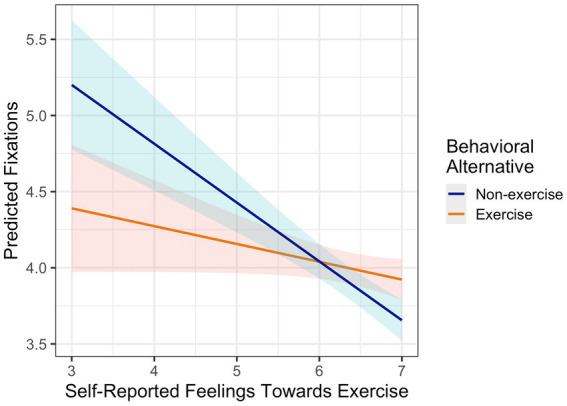
Predicting fixations on the exercise (orange) and non-exercise alternative (blue) with self-reported feelings towards exercise.

Automatic valuation of exercise, self-reported feelings towards exercise, and self-reported exercise volume were generally unrelated to the number of fixations on the exercise (see Table 2) and on the non-exercise alternative (see Table 3). Only the number of gaze fixations on the non-exercise alternative was slightly associated with self-reported feelings towards exercise (*b* = −0.27, 95% CI [−0.53, −0.00], *p* = 0.05). Figure 3 illustrates that more positive reported feelings towards exercise were not associated with more exercise fixations (orange line), but more negative reported feelings were associated with more fixations on the non-exercise alternative (blue line). These findings indicate the number of fixations was statistically informative for the behavioral choices in the task, but it was not associated with what participants typically like (automatic valuation and self-reported feelings towards exercise) or their usual behavior (self-reported exercise volume).

**Table 2:** Predicting exercise fixations with automatic valuation of exercise (Model A), self-reported feelings towards exercise (Model B) and self-reported exercise behavior (Model C) when making exercise-related choices.

	Model A			Model B			Model C		
	*b*	95% CI	*p*	*b*	95% CI	*p*	*b*	95% CI	*p*
(IC)	3.80	3.43, 4.16	<0.001	3.79	3.43, 4.16	<0.001	3.78	3.27, 4.29	<0.001
Choice [ex]	0.37	0.11, 0.63	0.01	0.38	0.12, 0.64	<0.01	0.33	0.09, 0.57	0.01
Aut. ex valuation	−0.16	−0.46, 0.13	0.27						
SR ex feelings				−0.17	−0.46, 0.12	0.25			
SR ex volume								−0.00, 0.00	0.55

CI = Confidence Interval; b = unstandardized regression estimate; IC = intercept; ex = exercise; Aut. = automatic; SR = self-reported.

**Table 3:** Predicting non-exercise fixations with automatic valuation of exercise (Model D), self-reported feelings towards exercise (Model E) and self-reported exercise behavior (Model F) when making exercise-related choices.

	Model D			Model E			Model F		
	*b*	95% CI	*p*	*b*	95% CI	*p*	*b*	95% CI	*p*
(IC)	4.88	4.49, 5.26	<0.001	4.87	4.48, 5.26	<0.001	4.75	4.24, 5.25	<0.001
Choice [ex]	−1.54	−1.78, −1.31	<0.001	−1.53	−1.77, −1.29	<0.001	−1.56	−1.80, −1.32	<0.001
Aut. ex valuation	−0.16	−0.42, 0.10	0.27						
SR ex feelings				−0.27	−0.53, −0.00	0.05			
SR ex volume							0.00	−0.00, 0.00	0.79

CI = Confidence Interval; b = unstandardized regression estimate; IC = intercept; ex = exercise; Aut. = automatic; SR = self-reported.

## 4. Discussion

This study examined situated processes and interindividual differences in gaze behavior in a sample of healthy individuals when confronted with a choice between two behavioral alternatives: to exercise or not to exercise. We found that individuals’ gaze behavior was associated with their in-the-moment choices, but not with their more general automatic affective valuation, their controlled evaluation of exercise, and not even with their self-reported exercise behavior. Findings suggest that individuals are more likely to focus on what they are about to choose in a single situation, but not what they usually like or do. Our results provide evidence that situated processes that arise from very specific stimulus configurations with behavioral alternatives can be independent of individuals’ more general preferences.

These findings partially support theoretical perspectives from dual-process models such as the ART (Brand and Ekkekakis, 2018) and the TEMPA (Cheval and Boisgontier, 2021; or the Automatic Affective Evaluations of Physical Activity model, to name another; Conroy and Berry, 2017) that situated and probably conflicting processes between behavioral alternatives need to become a greater focus of research when analyzing behavioral choices. After having established the intention, for example, to start an exercise routine, the resulting behavior is often an in-the-moment choice between behavioral alternatives. Individuals may experience conflicts thereby, because choices involve the desired behavior (e.g., exercise) and an alternative behavior that may be a barrier for engaging in the desired behavior (e.g., lying on the couch). Therefore, not only should the processes that drive someone towards the desired behavior (e.g., beliefs, goals) be analyzed, but also the processes that occur in a particular situation (i.e., situated processes) that prevent someone from engaging in that desired behavior.

As expected, individuals who reported to generally like and do exercise were more likely to choose the exercise alternative (65%) than the non-exercise alternative in the behavioral alternatives task. This fits well with the self-reported exercise volume of the present study sample. We had a fairly active sample with the middle 50% of participants reporting to have between 180 and 450 min of exercise per week (*M* = 358, *SD* = 283). Thus, the sum of the individual choices in the behavioral alternatives task seems to reflect general exercise preferences.

There was no automatic bias in first gaze to either the exercise or the non-exercise alternative. This neither supports assumptions of ART nor TEMPA. Based on TEMPA, there would have been a general automatic bias towards the non-exercise alternative due to an inherent universal bias toward effort minimization. Alternatively, ART would suggest automatic responses are learned through experiences and triggered when confronted with an exercise-related stimulus. Based on ART, participants would initially direct their gaze in line with their automatic valuation of exercise. However, those who had a more positive automatic valuation of exercise had no automatic bias towards the exercise alternative. This result could also be biased by the relatively active sample (due to the limited variance in the exercise volume variable). Another possible explanation for these findings is that the AMP is just a proxy for measuring automatic valuations and may not adequately represent the construct of automatic affective valuation of exercise, despite robust findings in other fields (Payne and Lundberg, 2014). Only one study to date has shown a medium size effect (*d* = 0.59) between the AMP score and exercise behavior (Antoniewicz and Brand, 2014). In particular, these authors showed that frequent fitness center exercisers exhibited more positive affective valuation of fitness center exercising than exercisers who preferred other exercise settings. In the present study, the AMP score was significantly, but only slightly (*r* = 0.20, *p* = 0.05) correlated with choice behavior and unrelated to self-reported exercise volume (*r* = 0.15, *p* = 0.12). This does not necessarily mean that the AMP has no validity, but the results obtained with the AMP should be interpreted cautiously on a more nuanced level. The present findings (a higher, albeit small, correlation between the AMP and choice behavior than with exercise volume) support Antoniewicz and Brand’s (2014) conclusion that automatic affective valuations may play a role in qualitative behavioral regulation (e.g., choice of exercise setting) rather than in quantitative behavioral regulation (i.e., exercise volume). Additionally, with the AMP, automatic valuations were not measured on a situational basis (i.e., for each choice situation). According to ART (Brand and Ekkekakis, 2018) automatic valuations of exercise arise and manifest themselves in situated decisions, meaning that automatic processes may vary depending on the situation at hand (e.g., the specific behavioral alternatives an individual faces). In the present study, however, affective valuation was measured only once with the AMP and thus may not be able to predict situated gaze behavior. This would require a tool that measures automatic valuations for each individual situation, which to our best knowledge does not yet exist.

As expected, first gaze was associated, albeit slightly, with the alternative chosen in that situation. This pattern of results is even more evident for fixations where participants directed their gaze on a specific location in the picture. These findings are in line with a large body of evidence on the gaze cascade effect, the tendency to look longer at stimuli that are eventually chosen (e.g., Onuma et al., 2017). Interestingly, similar to first gaze, the number of fixations were not associated with the assessed interindividual differences. For example, active individuals did not look longer at the exercise stimuli than inactive participants. These results seem to contradict previous findings from exercise psychology which have demonstrated an attentional bias towards exercise for active individuals (e.g., Berry et al., 2011; Cheval et al., 2020). However, in comparison to the study here, participants in previous studies were not forced to make a choice. There is research showing that attentional processes are more strongly influenced by the task itself (i.e., the goal of the decision: to choose what you want vs. what you do not want) than individual preferences (van der Laan et al., 2015). Our findings support this by showing that the task (to make a choice) and the specific alternatives presented in each situation (i.e., the presented behavioral alternatives) were associated with gaze behavior but not with individual preferences or behaviors. Hence, this lends support for the importance of situated processes emphasized in theoretical perspectives from dual-process framework (Brand and Cheval, 2019; Rhodes et al., 2019).

Although an individual may report liking exercise, certain features of an alternative behavior may drive the individual to choose the alternative over exercise. This is well in line with the idea of an inner conflict. Even if someone generally likes to exercise, but the couch seems more attractive in that very situation, an internal conflict arises. More attention may be on the non-exercise alternative, which increases the likelihood that the alternative behavior will be chosen. This suggests that in-the-moment individuals are confronted with the decision to exercise, additional situated processes may influence the decision. Thus, our results support the assumption that attentional processes may play an active role in constructing choice behavior above and beyond general preferences (Orquin and Mueller Loose, 2013).

Assuming that the present findings are robust and replicable, this could imply that neither an inherent nor a learned automatic bias toward exercise or a sedentary alternative can sufficiently explain behavioral choices. This challenges assumptions of TEMPA regarding a negative automatic bias towards exercise and some predictions of ART regarding a learned automatic association of exercise. On the other hand, a more fundamental assumption of dual process models can be supported. We found that processes that take place in-the-moment of choice play an active role in constructing the choice. This is consistent with the assumption of a continuous interaction between situated automatic-affective and reflective processes until a choice is reached (Brand and Ekkekakis, 2021). Further refinement would be needed with respect to assumptions about the interplay between psychological states and traits. The present study suggests that individuals bring some inherent general trait-like preferences (e.g., liking exercise) into a situation, but these general preferences may operate independently of state-like situated processes (e.g., the affective state).

In line with current perspectives of exercise behavior change (Rhodes et al., 2019), exercise interventions largely focused on interindividual preferences or differences may fail at long-term behavior change because they neglect the role of situated processes and competing behavioral tendencies (e.g., the appeal of a non-exercise behavioral alternative). Empirical studies focused on interindividual difference – such as perceived autonomy, competence, or relatedness – may explain behavior change, but intervention focused on these variables fail to result in sustained behavior change (Chevance et al., 2019; Compernolle et al., 2019; Ntoumanis et al., 2021). In order to improve exercise interventions, situational features such as attention to specific behavioral alternatives should be considered in addition to interindividual differences, e.g., in expectations and goals.

### 4.1. Limitations and future directions

While the study had several strengths (e.g., capturing processes in-the-moment of choice, using generalized mixed models), some limitations need to be considered. In the present study, hypothetical scenarios were used as a proxy for situated decision-making. Future studies should examine how the present results unfold in real life. One way to investigate situated processes in real life decisions could be the use of ecological momentary assessment (EMA), which can capture time-varying factors and intraindividual fluctuations (e.g., Dunton, 2017). EMA has been shown to be a feasible way to measure exercise behavior and motivation in real-time and naturalistic settings (Maher et al., 2018; Reichert et al., 2022). Studies using this technique already yielded reliable associations between momentary affective states and physical activity behavior (Liao et al., 2015). However, a randomized-controlled trial that investigated the effects of an intervention on controlled processes (goal setting) on daily physical activity levels failed to demonstrate a significant effect. Instead, these results revealed substantial individual variability, suggesting that other processes may play a role in promoting or hindering physical activity (Utesch et al., 2022). Automatic processes could be one of those variables. However, there is yet to be a tool that can capture automatic processes - such as those measured with the AMP - on a momentary basis. As an alternative, quick implicit measures such as the brief implicit association test (Sriram and Greenwald, 2009) or eye-tracking (Peng et al., 2021) could be modified for mobile devices.

Despite the use of a within-subject design, the present study is unable to conclude causal relationships. Future work is needed to understand whether exercise-related choice preferences can be influenced by experimentally manipulating attentional processes. Moreover, as the study sample consisted mostly of university students, generalizability is limited. It is possible that because many participants were enrolled in a physical activity focused program, this may have caused the bias toward the exercise alternative. The behavioral alternatives task appears to successfully assess a tendency of individuals to choose exercise, but it is important to note that the odds found in this study (preference for the exercise alternative) may not reflect the general population. This calls for replication studies with more heterogenous and larger sample sizes.

In addition, this task had relatively few trials compared to other eye-tracking or experimental studies (van der Laan et al., 2015). However, the focus of the present task was to examine processes within trials (choices) and not on an overall general score across all trials. Modeling both, participants and stimuli as random effects helped to increase the robustness of statistical analytics beyond the specific stimuli used (Westfall et al., 2014). However, if the focus of a study would be to examine a general preference across trials, more trials would certainly be needed.

The unique features of the computerized behavioral alternatives task – such as modeling single situated choices on different levels and the use of eye-tracking as a process-tracing method – open up possibilities to test hypotheses derived from exercise psychology theories. For example, it could be studied whether limited self-control alters the interplay of automatic and controlled processes or whether changing the affective experience during the behavior (e.g., Jones et al., 2020; Timme and Brand, 2020) influences exercise-related information processing. Furthermore, it would be interesting to investigate how stable these processes are and whether situational influences (such as exercising before the task) would render, for example, sedentary activities more attractive.

In terms of practical implications, our findings suggest that, for example, personal trainers should consider that situational factors (e.g., the specific behavioral alternatives) influence whether or not individuals follow an exercise program, probably quite independently of their more general beliefs and preferences.

## 5. Conclusion

Previous studies and interventions for exercise behavior change have largely focused on interindividual differences in automatic and controlled processes. This study provided partial support for dual-process theories in exercise psychology. We found that interindividual differences in general exercise preferences (i.e., automatic-affective valuation, controlled evaluation and exercise behavior) are related to the choice behavior among concrete behavioral alternatives (exercise vs. non-exercise). However, situated gaze behavior in these choice situations does not follow these interindividual preferences, but rather depends on the specific available behavioral alternatives. This implies that situated processes may augment interindividual differences in automatic and controlled (e)valuations of exercise when it comes to exercise-related choices. The importance of situated processes in behavior change has been neglected by most exercise psychology theories so far, and thus may be an important missing piece in understanding the processes underlying exercise motivation.

## Data availability statement

The datasets presented in this study can be found in online repositories. The names of the repository/repositories and accession number(s) can be found at: https://osf.io/ubrj7/.

## Ethics statement

Ethical review and approval were not required for the study on human participants in accordance with the local legislation and institutional requirements. The participants provided their written informed consent to participate in this study.

## Author contributions

MR, RB, and ST developed the experimental design and carried out the data collection. ST performed the data analysis and wrote the first draft of the manuscript. MR and RB edited the manuscript. All authors contributed to the article and approved the submitted version.

## Funding

During the analysis and manuscript preparation, ST was supported by the German Academic Scholarship Foundation. Open access costs were funded by the Deutsche Forschungsgemeinschaft (DFG, German Research Foundation) – project number 491466077. This funding body had no role in the study design, analysis, interpretation of findings, or writing of the manuscript.

## Conflict of interest

The authors declare that the research was conducted in the absence of any commercial or financial relationships that could be construed as a potential conflict of interest.

## Publisher’s note

All claims expressed in this article are solely those of the authors and do not necessarily represent those of their affiliated organizations, or those of the publisher, the editors and the reviewers. Any product that may be evaluated in this article, or claim that may be made by its manufacturer, is not guaranteed or endorsed by the publisher.

## Footnotes

^1^ https://osf.io/ubrj7/

^2^ pixabay.com
